# Supplementary figures and images for: The type IV pilus chemoreceptor PilJ controls chemotaxis of one bacterial species towards another
Source: PLoS Biol. 2024 Feb 13;22(2):e3002488. doi: 10.1371/journal.pbio.3002488 (PMC10896506; doi:10.1371/journal.pbio.3002488)

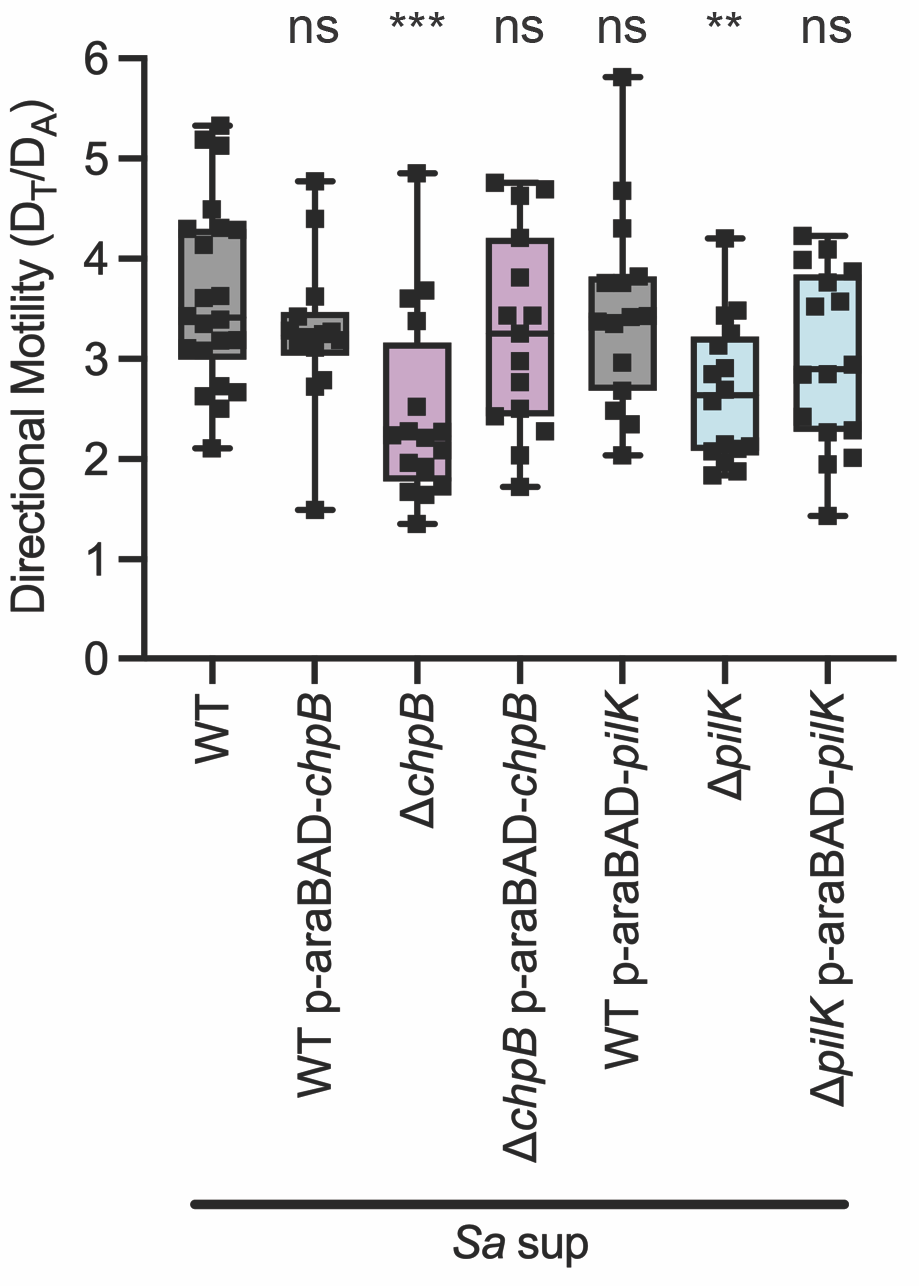

Supplement: S1 Fig — Directional motility towards S. aureus secreted factors of wild type, ΔpilK, and ΔchpB with and without complementing plasmids carrying arabinose-inducible copies of pilK or chpB. Complemented strains were induced with 0.2% arabinose; however, phenotypes were the same in the absence of induction. Directional motility for at least 4 biological replicates, each containing a minimum of 3 technical replicates are shown. Statistical significance was determined with a one-way ANOVA followed by Dunnett’s multiple comparisons test. *** indicates p < 0.001; ** indicates p < 0.01; ns indicates no statistically significant difference. The underlying data can be found in S1 Data. (TIFF) [file pbio.3002488.s001.tiff]

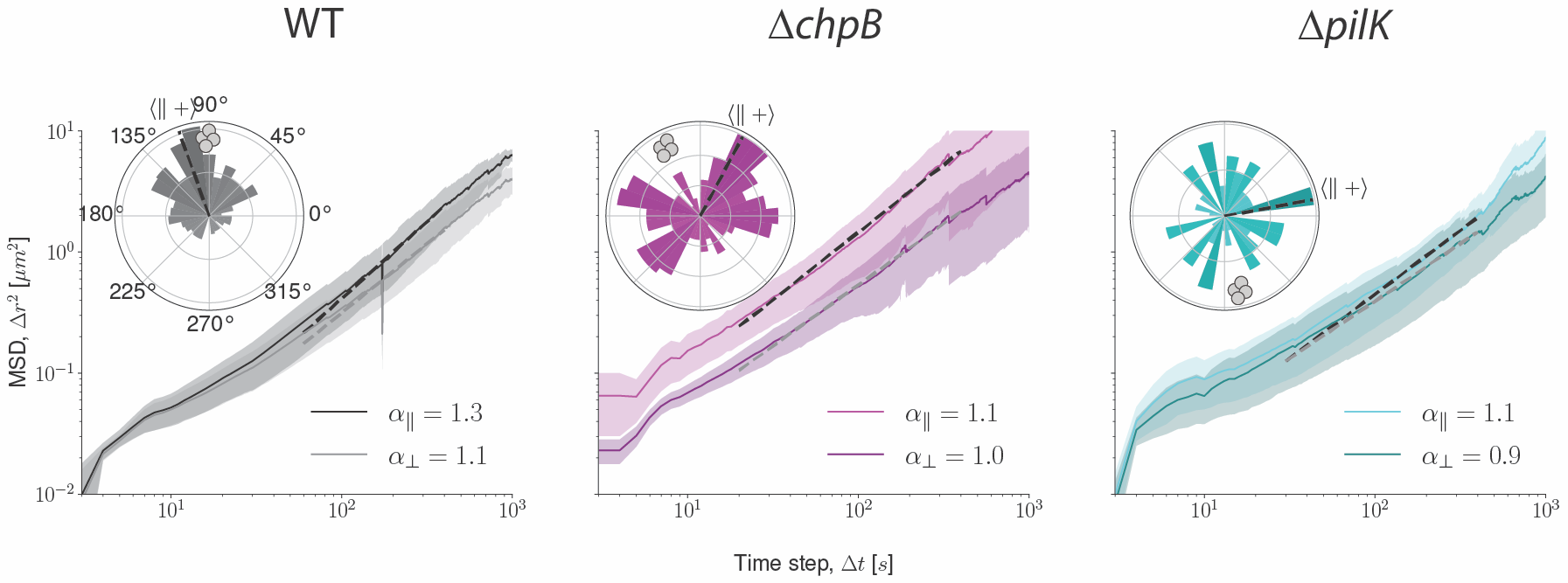

Supplement: S2 Fig — MSDs for the parallel (||) and perpendicular (⊥) directions of wild type, ΔchpB, and ΔpilK across different time steps (lag times, Δt). The anomalous diffusion exponent (α) for each MSD is shown. Insets show the corresponding rose graphs from Fig 2B. The underlying data can be found in S2–S4 Data. (TIFF) [file pbio.3002488.s002.tiff]

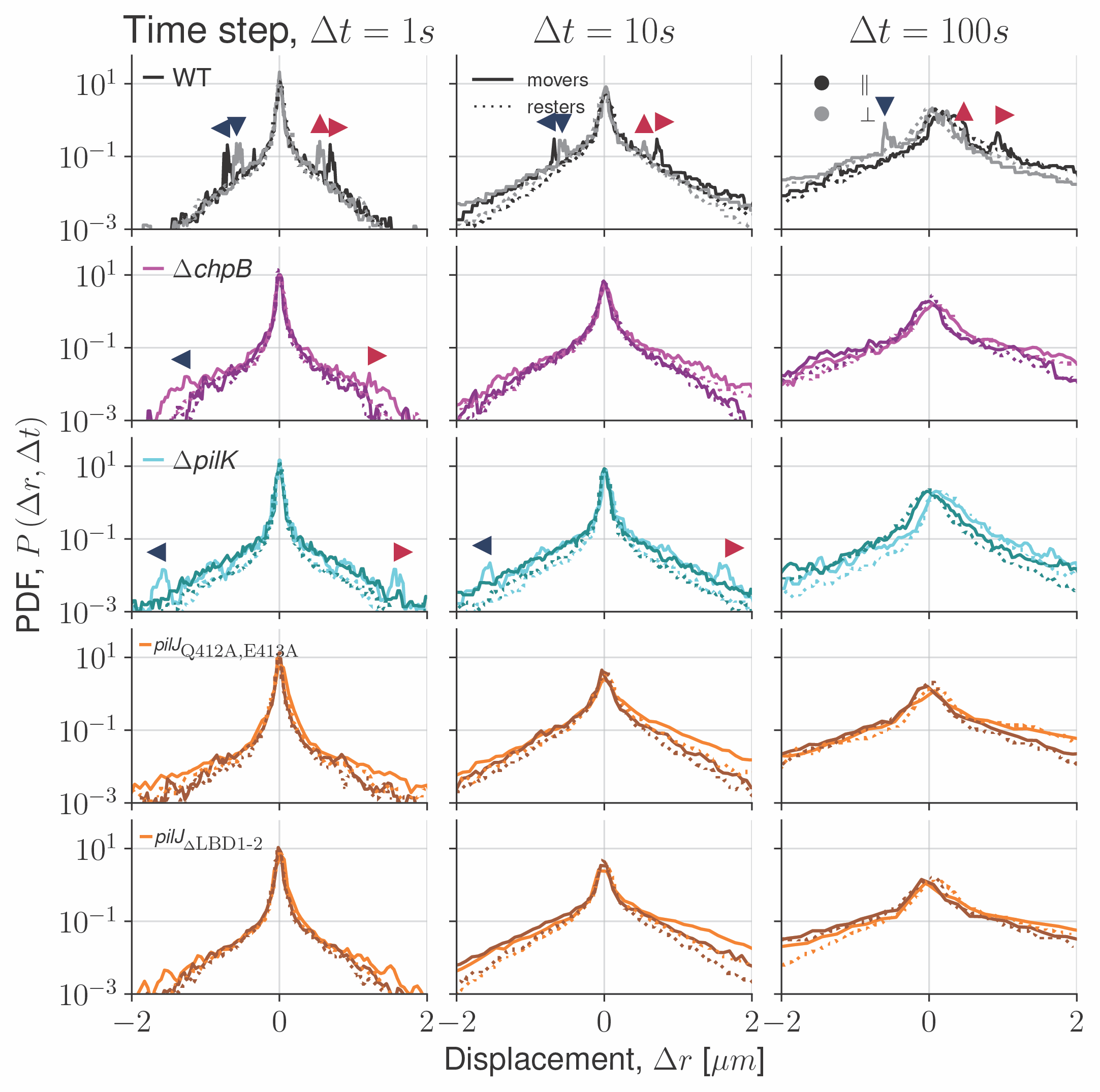

Supplement: S3 Fig — Step size distributions for each P. aeruginosa strain are displayed by distance of displacement (Δr) of cells. Step size PDFs are shown for movers (solid lines) and resters (dotted lines) in the parallel (darker lines, ||) and perpendicular (lighter lines, ⊥) directions. Step sizes for each P. aeruginosa strain were calculated from cell trajectories with a 1-second (left), 10-second (middle), and 100-second (right) time step (Δt). Right- and left-facing triangles (movers, ||+ and ||−, respectively) and up- and down-facing triangles (movers, ⊥+ and ⊥−, respectively) highlight the nonzero sharp-shoulder peak step size, when present. The underlying data can be found in S2–S6 Data. (TIFF) [file pbio.3002488.s003.tiff]

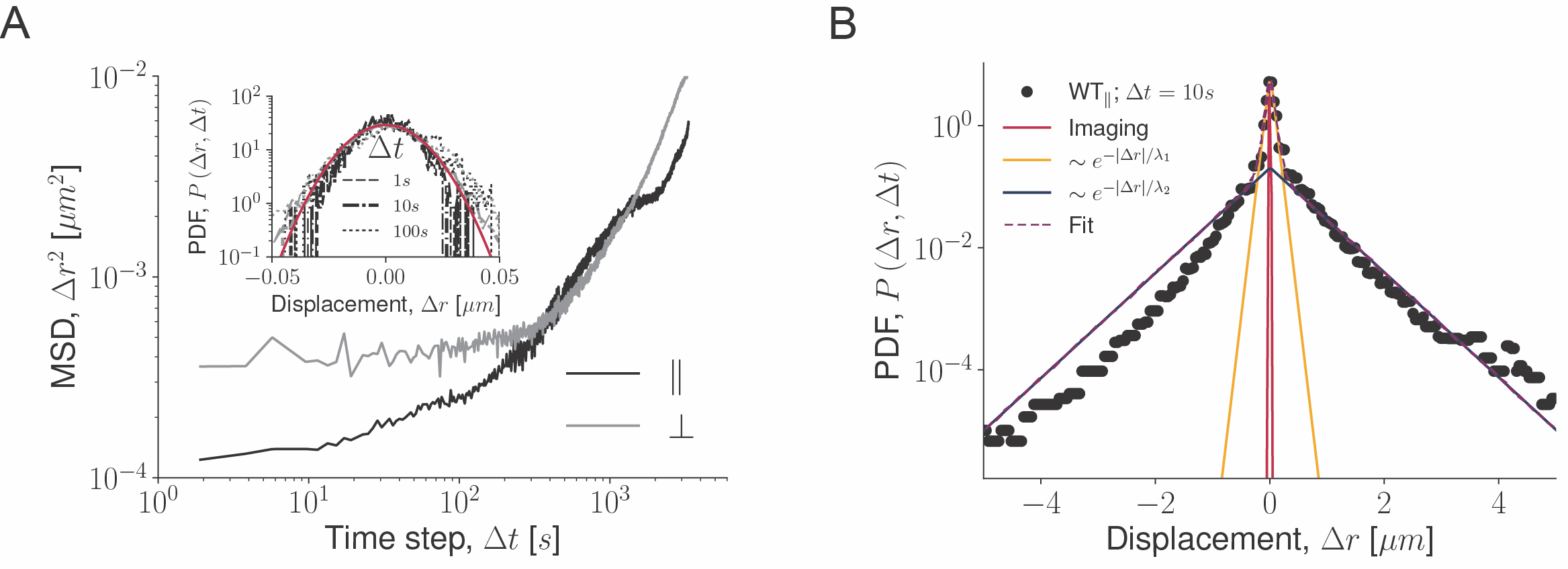

Supplement: S4 Fig — (A) MSDs for the parallel (||) and perpendicular (⊥) directions for dust particles used to measure noise in the imaging. Inset shows the particle-displacement PDF. The PDF is a narrow noise peak that is fit to a Gaussian distribution (solid red line) but is nondiffusive, as it does not broaden in time. (B) PDF of the total cell step displacements (Δr), regardless of principal direction, for wild type cells at a time step of Δt = 10 seconds (black dots). The PDF is composed of a narrow peak of small displacements (jiggling) and long tails of large-but-rare displacements. The narrow peak cannot be explained by imaging uncertainty (solid red curve) and is better described by a Laplace distribution (Eq 7; solid yellow line), as are the long tails (solid dark blue line). The underlying data can be found in S1 and S7 Data. (TIFF) [file pbio.3002488.s004.tiff]

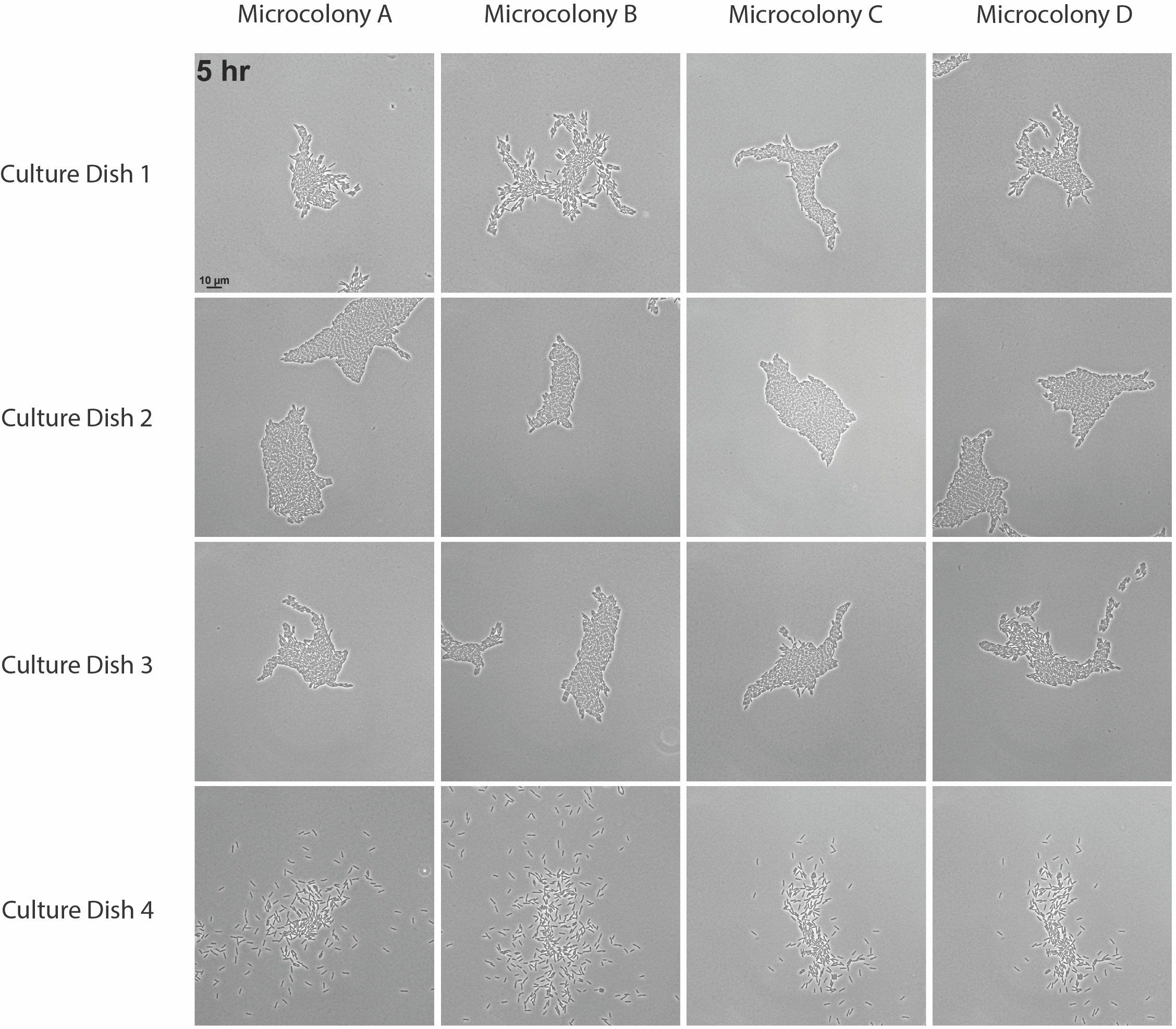

Supplement: S5 Fig — Separate ΔpilK cultures plated onto 4 individual experimental dishes and 4 fields of view in each culture dish were simultaneously imaged at 5 hours postinoculation. Agarose pads were made from the same media and dried under the same conditions at the same time. A range of motility phenotypes are seen between all microcolonies imaged. (TIFF) [file pbio.3002488.s005.tiff]

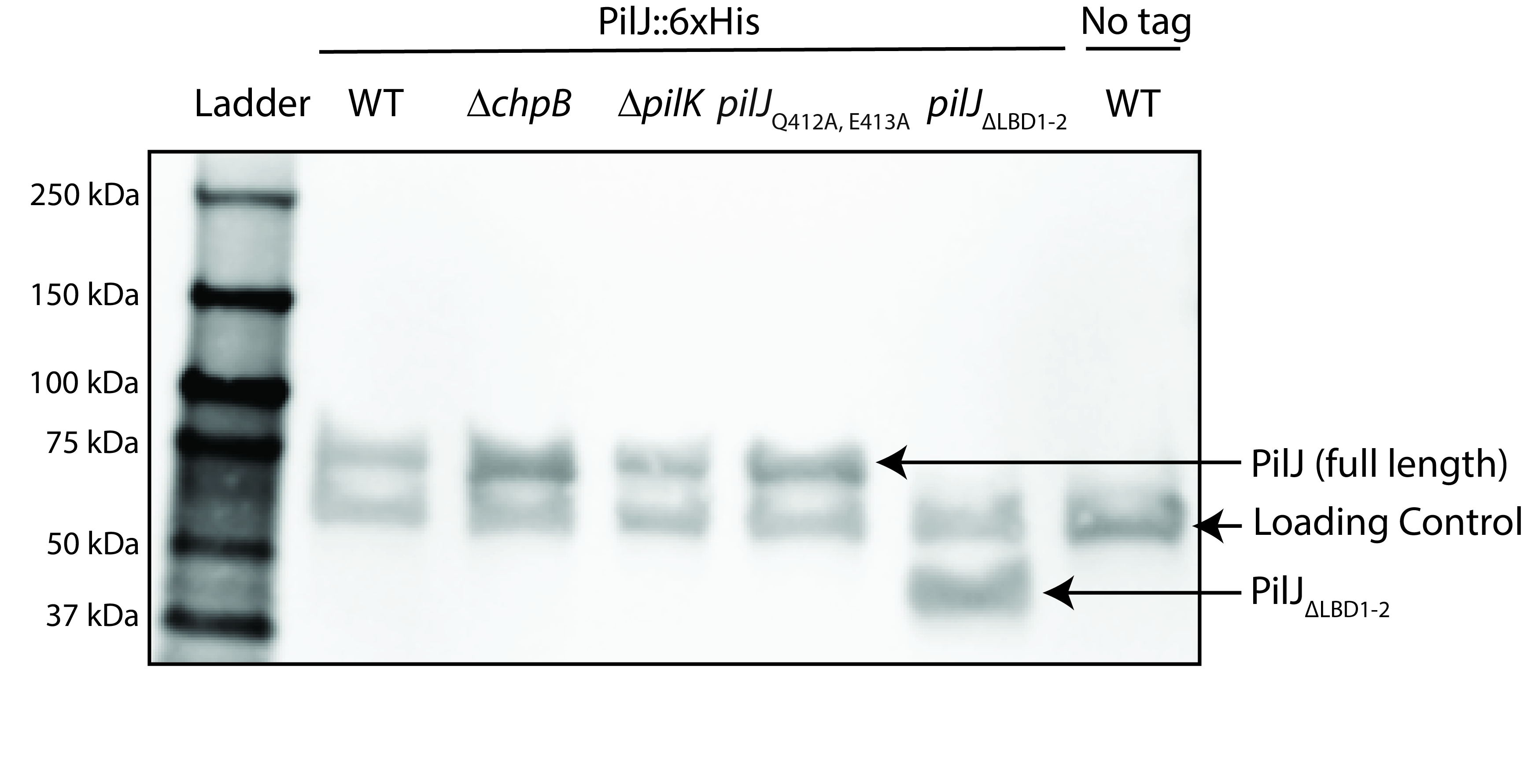

Supplement: S6 Fig — Western blot of His-tagged WT or mutant PilJ protein derived from P. aeruginosa. An untagged protein sample from P. aeruginosa WT was used as a control. Full-length PilJ is expected to be 75 kDa, while the PilJ protein lacking the LBDs is expected to be 46 kDa. Detection was performed for PilJ::6xHis alone (A) and for PilJ::6xHis with a loading control (B). (TIF) [file pbio.3002488.s006.tif]

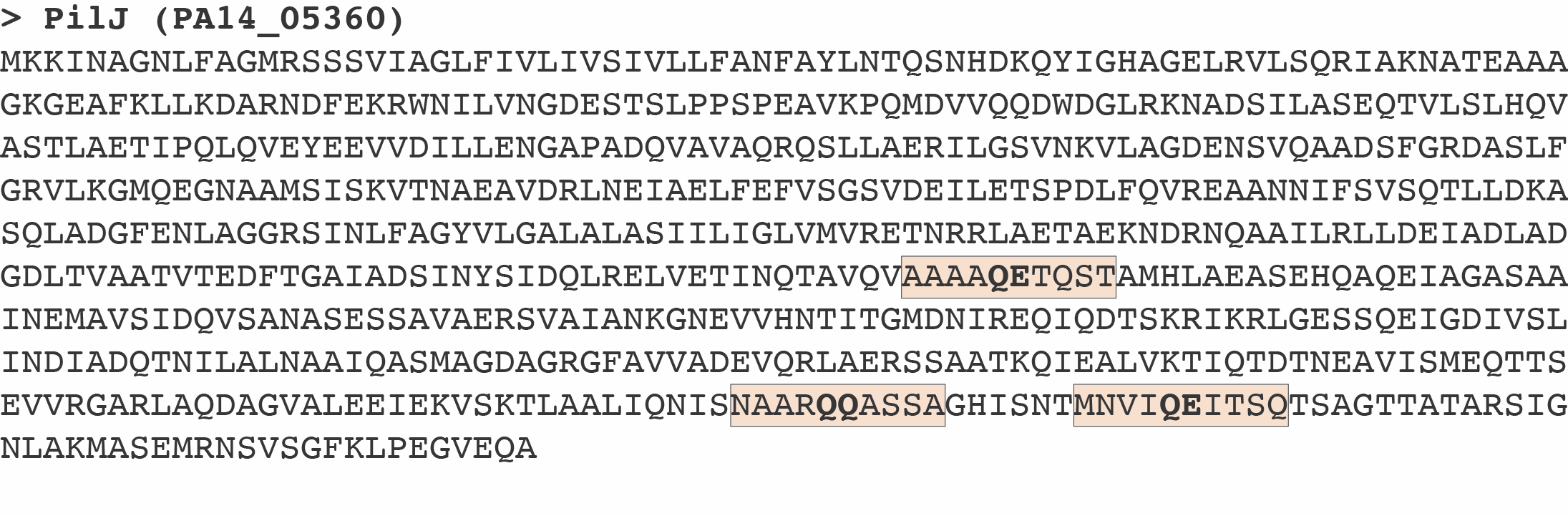

Supplement: S7 Fig — Amino acid sequence of P. aeruginosa PA14 PilJ (PA14_05360) with conserved MCP methylation motifs highlighted in pale orange and predicted methyl modification glutamate/glutamine residue pairs in bold. (TIFF) [file pbio.3002488.s007.tiff]

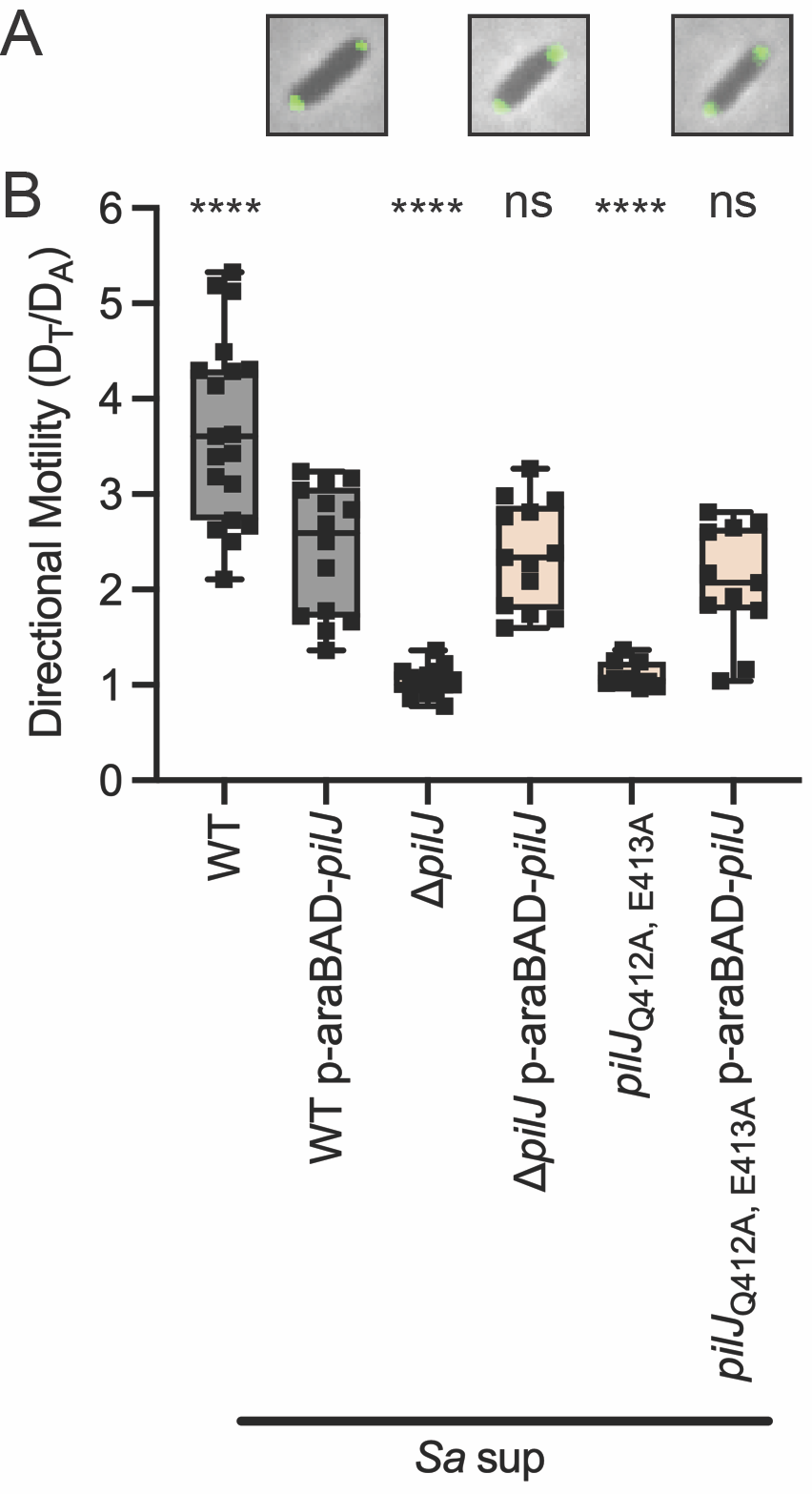

Supplement: S8 Fig — (A) Representative P. aeruginosa cells with bipolarly localized GFP-tagged PilJ. (B) Directional motility towards S. aureus secreted factors of wild type or pilJQ412A, E413A with and without complementing plasmids carrying arabinose-inducible copy of wild type pilJ. Complemented strains were induced with 0.2% arabinose; however, phenotypes were the same in the absence of induction. Directional motility for at least 3 biological replicates, each containing a minimum of 3 technical replicates are shown, and statistical significance was determined with a one-way ANOVA followed by Dunnett’s multiple comparisons test to compare each strain to wild type P. aeruginosa carrying p-araBAD-pilJ. **** indicates p < 0.0001; ns indicates no statistically significant difference. The underlying data can be found in S1 Data. (TIFF) [file pbio.3002488.s008.tiff]

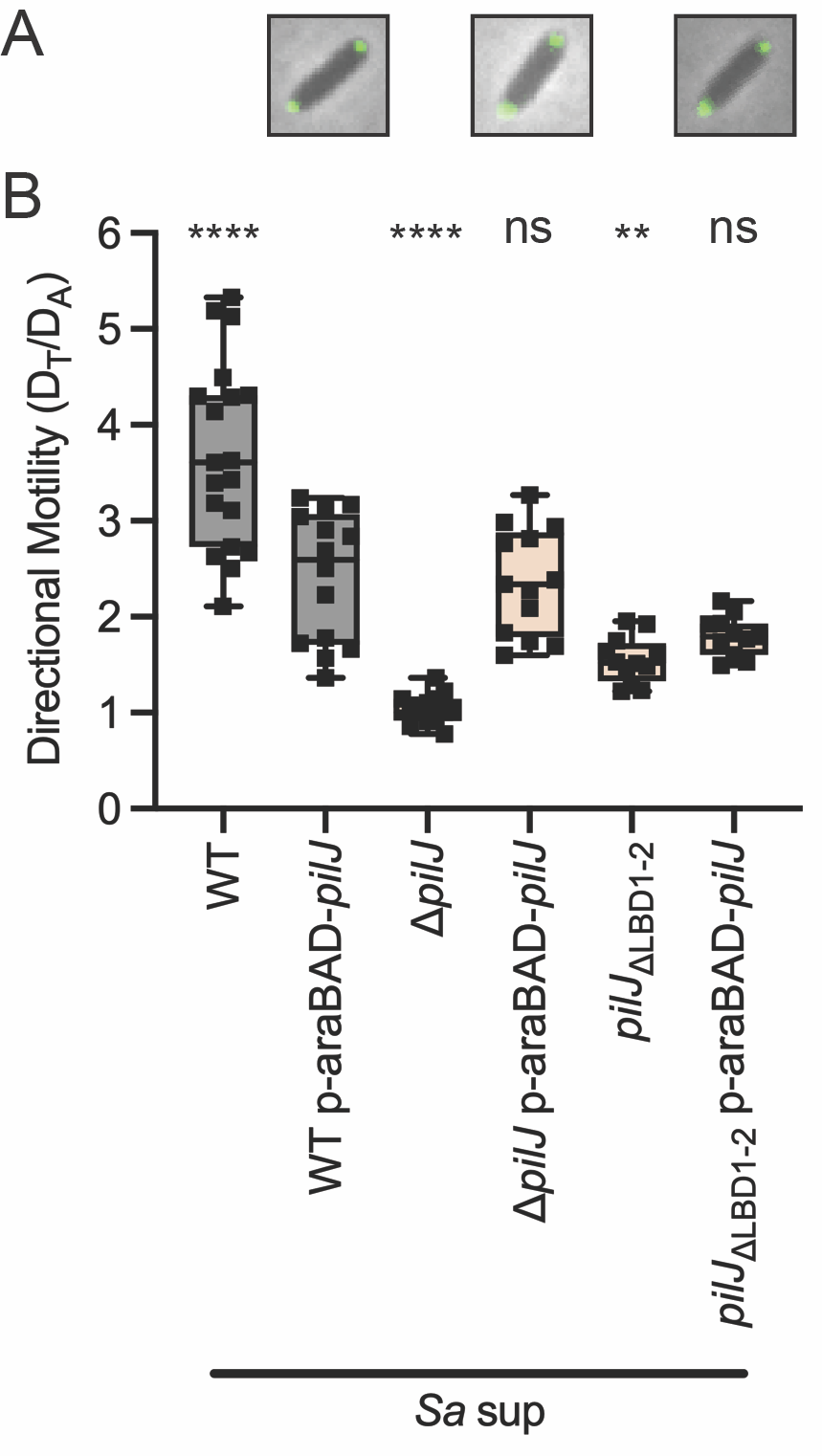

Supplement: S9 Fig — (A) Representative P. aeruginosa cells with bipolarly localized GFP-tagged PilJ. (B) Directional motility towards S. aureus secreted factors of wild type or pilJΔLBD1-2 with and without complementing plasmid carrying arabinose-inducible copy of wild type pilJ. Complemented strains were induced with 0.2% arabinose; however, phenotypes were the same in the absence of induction. Directional motility for at least 3 biological replicates, each containing a minimum of 3 technical replicates are shown, and statistical significance was determined with a one-way ANOVA followed by Dunnett’s multiple comparisons test to compare each strain to wild type P. aeruginosa carrying p-araBAD-pilJ. **** indicates p < 0.0001; ** indicates p < 0.01; ns indicates no statistically significant difference. The underlying data can be found in S1 Data. (TIFF) [file pbio.3002488.s009.tiff]

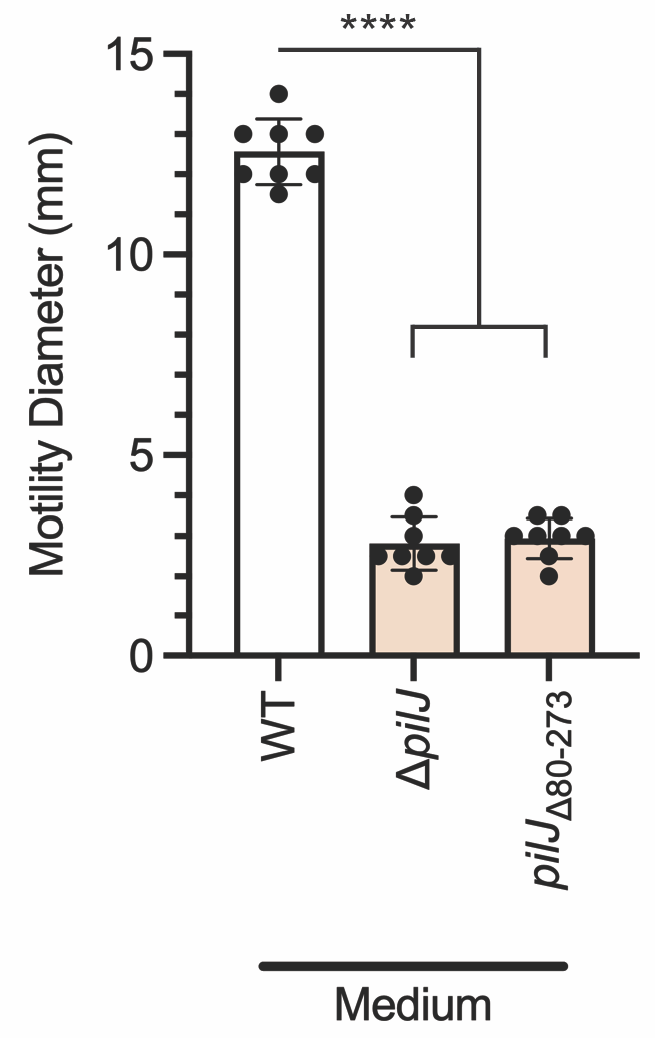

Supplement: S10 Fig — Twitching motility diameters of P. aeruginosa wild type, ΔpilJ, and a pilJ mutant lacking amino acids 80-273 (pilJΔ80-273). Macroscopic motility measurements are shown for 2 biological replicates, each containing 4 technical replicates, and statistical significance was determined with a one-way ANOVA followed by Dunnett’s multiple comparisons test. **** indicates p < 0.0001. The underlying data can be found in S1 Data. (TIFF) [file pbio.3002488.s010.tiff]

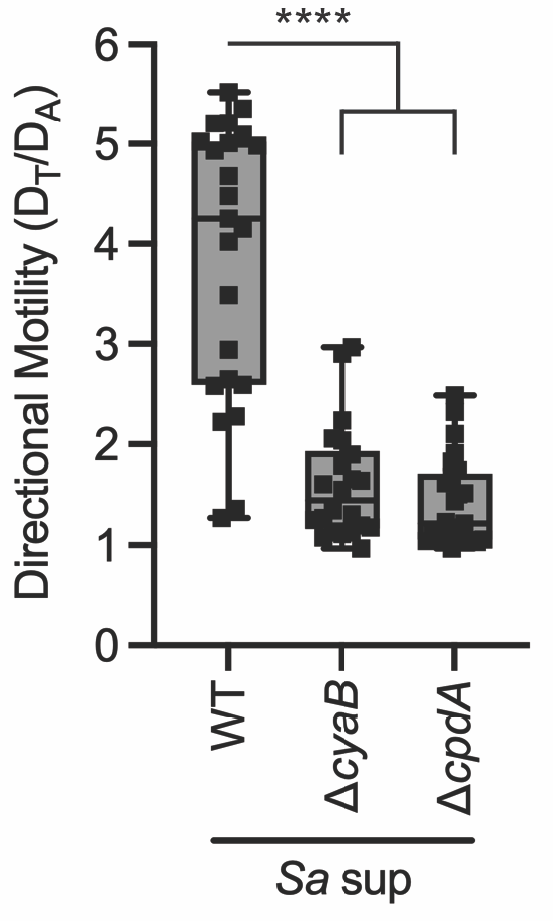

Supplement: S11 Fig — Directional motility towards S. aureus secreted factors of ΔcyaB and ΔcpdA. At least 3 biological replicates, each containing a minimum of 3 technical replicates are shown, and statistical significance was determined with a one-way ANOVA followed by Dunnett’s multiple comparisons test. **** indicates p < 0.0001. The underlying data can be found in S1 Data. (TIFF) [file pbio.3002488.s011.tiff]
