# Supplementary material for: The type IV pilus chemoreceptor PilJ controls chemotaxis of one bacterial species towards another
Source: PLoS Biol. 2024 Feb 13;22(2):e3002488. doi: 10.1371/journal.pbio.3002488 (PMC10896506; doi:10.1371/journal.pbio.3002488)
Supplement: S1 Raw Image — (PDF) [file pbio.3002488.s042.pdf]

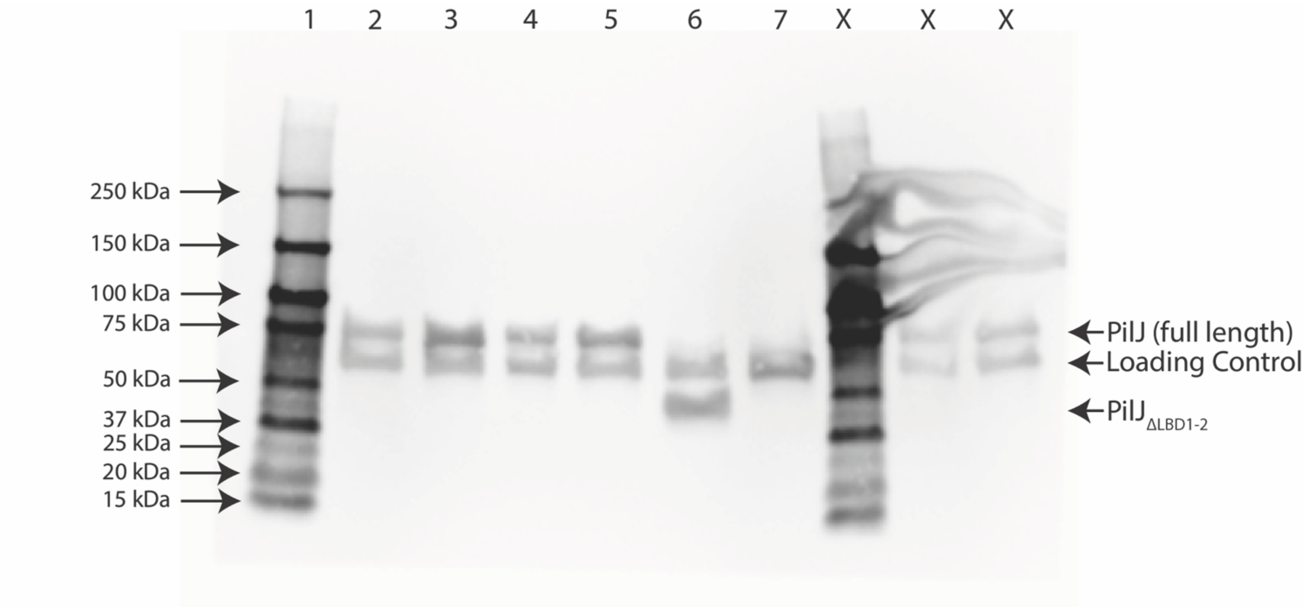

Original blot image for S6 Fig.
Lanes 1-7 are shown in the final supplemental figure.
The final image was captured with an Azure Sapphire Imager.

Description of experimental sample in each lane:
Lane 1 = Molecular weight ladder. Molecular weights for each band are labeled to the left of the blot.
Lane 2 = Wildtype PilJ::6xHis protein from *P. aeruginosa* PA14 wildtype.
Lane 3 = Wildtype PilJ::6xHis protein from *P. aeruginosa* PA14 Δ*chpB*.
Lane 4 = Wildtype PilJ::6xHis protein from *P. aeruginosa* PA14 Δ*pilK*.
Lane 5 = $PilJ_{Q412A,\ E413A}$::6xHis protein from *P. aeruginosa* PA14 $pilJ_{Q412A,\ E413A}$.
Lane 6 = $PilJ_{\Delta LBD1\text{-}2}$::6xHis protein from *P. aeruginosa* PA14 $pilJ_{\Delta LBD1\text{-}2}$.
Lane 7 = Wildtype PilJ protein (untagged) from *P. aeruginosa* PA14 wildtype.
Lanes labeled with “X” were not included in the final figure.

The band for full length PilJ is around 75 kDa in lanes 2-5. The band for $PilJ_{\Delta LBD1\text{-}2}$ is around 46 kDa in lane 6.
The band around 65 kDa in lanes 2-7 is the loading control.
