# Supplementary material for: The type IV pilus chemoreceptor PilJ controls chemotaxis of one bacterial species towards another
Source: PLoS Biol. 2024 Feb 13;22(2):e3002488. doi: 10.1371/journal.pbio.3002488 (PMC10896506; doi:10.1371/journal.pbio.3002488)
Supplement: Attachment — Submitted filename: Yarrington_ResponsetoReviewers_PLOSBiology_Revision.pdf [file pbio.3002488.s043.pdf]

Dear Dr. Jauregui and Academic Editor,

We thank you very much for taking the time to read through our manuscript and prior reviews and for providing helpful suggestions to improve the article. Please find our responses, in bold, to each comment below and thank you for consideration of this article.

EDITED COMMENTS FROM THE ACADEMIC EDITOR:
Based on the reviews of reviewers #1 and #3 and the authors' responses, I am very much in favour of this manuscript for PLOS Biology. The authors also respond very convincingly to the more critical comments of reviewer #2.

> **We thank the editor for thoroughly reading the manuscript with its previous reviews and for providing feedback on both the manuscript and responses to the reviewers.**

The elucidation of the behavioural mechanism that results in chemotaxis towards PSMs is very interesting, novel and of broad interest, however, the discussion of this finding is not very elaborate. The authors should discuss, compare and contrast the behavioural mechanism that results in chemotaxis in *P. aeruginosa* towards PSMs to the regulation of flagella-based motility.

> **We thank the reviewers for this excellent suggestion. In the revised manuscript, we have added a discussion on similarities and differences between regulation of flagella and pilus-mediated chemotaxis systems. Please see lines 577-626 in the clean copy of the revised article for these changes.**
